# Portable and Low-Cost Respirometric Microsystem for the Static and Dynamic Respirometry Monitoring of Compost

**DOI:** 10.3390/s19194132

**Published:** 2019-09-24

**Authors:** Juliette F. Bermudez, Juan F. Saldarriaga, Johann F. Osma

**Affiliations:** 1 Department of Civil and Environmental Engineering, Universidad de los Andes, Bogota 1100111, Colombia; jf.bermudez12@uniandes.edu.co (J.F.B.); jf.saldarriaga@uniandes.edu.co (J.F.S.); 2 CMUA. Department Electrical and Electronic Engineering, Universidad de los Andes, Bogota 1100111, Colombia

**Keywords:** compost, oxygen sensors, microfluidics system, respirometry

## Abstract

Composting is considered an option for the disposal of organic waste; however, the development of portable and low-cost systems for its monitoring is of high interest. Therefore, in this study, respirometric microsystems were designed and tested including two integrated oxygen sensors for the measurement of compost samples under static and dynamic conditions with high portability and ease of use. The cost of each sensor was calculated as 2 USD, while the cost of the whole respirometric microsystem was calculated as 6 USD. The electronic system for real-time monitoring was also designed and implemented. The designed systems were tested for over 6 weeks for the determination of compost quality using real samples. The respirometric microsystem was compared to a commercial respirometry system and a standard laboratory test using hierarchical analysis which included costs, portability accuracy, analysis time, and integration of new technologies. The analysis showed a global score of 6.87 for the respirometric microsystem compared to 6.70 for the standard laboratory test and 3.26 for the commercial system.

## 1. Introduction

One-third of the world’s food production is discarded every year [1,2]. This implies different types of environmental, social, and economic challenges, specifically in developing countries where the proportions of food waste (FW) in municipal waste are high [3,4,5,6]. Conventionally, FW is disposed in landfills or incinerated [7]; however, some countries are considering more sustainable methods for waste management, such as composting. [7,8]. Composting is the result of an aerobic biodegradation process of organic solid waste [9]. Its main use is as soil amendment and nutrient supply for plants and to increase the soil carbon stock [10]. Also, composting has other associated benefits, such as the profitability and the conversion of waste into value-added products. Due to the fact of these characteristics, composting has gained great popularity as an effective management strategy of FW and has been supported by several entities [7]. For instance, the FAO (Food and Agriculture Organization of the United Nations) recommended the use of composting to reduce the amount of waste in landfills, mitigate environmental impacts, and reduce the effects of FW [11]. In addition, the Environmental Protection Agency (EPA) of the United States declared that composting reduces methane emissions in landfills and reduces its carbon footprint [12]. 

Although composting has many benefits, the technification of the composting production process to increase its efficiency and guarantee its quality is needed [8]. The parameters related to the maturity and stability of composting must be measured to achieve this goal. A mature compost has to be suitable for its use as a fertilizer of plants or to improve phytotoxicity problems [8,13]. Stability is related to the degree of decomposition of biodegradable organic matter and, also, has an indirect relationship with the biological activity of a sample [8]. The maturity and stability of composting is evaluated by monitoring variables such as humidity, temperature, pH, nutrient content, carbon/nitrogen ratio (C/N), and oxygen consumption, among others [14,15]. 

Several studies considered oxygen consumption as a reliable variable for evaluating the development of composting, due to the fact of its correlation with the metabolic potential of compost [8,16,17,18], especially because oxygen is directly responsible for the oxidation of organic matter [19]. This can be measured through respirometry, a widely used and recognized technique for measuring the oxygen consumption of microorganisms [20,21]. This technique includes different methods that can be classified as static or dynamic. In static methods, a test is performed in the absence of air flow; while in dynamic methods, there is a continuous presence of air flow during tests [8,22,23]. The advantage of these methods is the high sensitivity to temperature changes and variations in the composition of the material [24,25]. However, there are important limitations for its use in composting at small and medium scales, due to the fact of their non-portable and ex-situ characteristics that are associated with the increase of costs and difficulties in the shipment of samples to laboratories. In addition, the use of such techniques requires trained manpower throughout the process [9,25,26].

Integrated sensors have been developed to overcome limitations such as cost, ease of use, and portability in different environmental studies [27,28], including air quality monitoring [29,30,31], control of automobile exhaust emissions, fuel consumption, and the control of chemical and biological processes [32,33]. The use of integrated sensors is an engaging tool for diagnosis due to the fact of their high sensitivity, short analysis time, cost-effectiveness, and potential of miniaturization [34,35,36,37]. In particular, oxygen sensors have been used to monitor the respirometry or uptake of oxygen by mammalian cells in biolabs, as a direct indicator for cell metabolism [38].

The use of microfluidics that integrate sensors, as a general platform for the manipulation, storage, and maintenance of the samples, while allowing for a more homogeneous environment for sensor measurements, has raised attention over the past few years. Portability limitations can be overcome with the use of microfluidics due to the fact of their ease of transport for samples, the possibility to mix reagents or the presence of reaction chambers inside confined microstructures with a minimum volume requirement of reagents [28,39,40]. In recent years, there some studies have been published on microfluidic chips with integrated oxygen detection. Molter et al. worked on a microfluidics system that allowed the measurement of single cells. This system contained glass microwells, while measurement took place thanks to a microscope with a highly sensitive camera [41]. Recently, Buenge et al. developed a microfluidic chip to determine the oxygen consumption rate of cells by measuring the concentration of dissolved oxygen. The microfluidics chamber was composed of a 200 μm × 2.5 mm × 12.3 mm chamber, and the chip contained an electric heater and a temperature sensor on the bottom [38].

In this paper, two different designs of integrated oxygen sensors were evaluated in terms of their electrical response versus oxygen concentration, cost analysis, and hierarchical analysis processes. A low-cost respirometric microsystem was designed including the best performing integrated oxygen sensor, a microfluidics system that included a sample chamber, the necessary microchannels that connected the different elements, and the electronic system for the real-time measurements. The respirometric microsystem was evaluated using samples of a compost process under static conditions over six weeks and dynamic conditions during the last stage of the compost process. Another hierarchical analysis process was carried out to study the viability of the manufactured respirometric microsystem and was compared to two available measuring methods, the commercial Ecozone System and a standard laboratory test of titration.

## 2. Materials and Methods

### 2.1. Reagents

Polymethylmethacrylate (PMMA) slides, methylene chloride, and the microscope slides were obtained at the local market; 99.9% lead pellets and 99.9% platinum wire were purchased from Kurt J. Lesker (Jefferson Hills, PA, USA); 1 inch double-sided tape and 1 inch copper tape were acquired from 3M (USA), ultra-high purity (>99.9%) nitrogen was purchased from Linde (Bogota, Colombia), 99.9% and potassium hydroxide (KOH) and 99.9% hydrofluoric acid (HF) were obtained from Sigma–Aldrich (Saint Louis, MO, USA). 

### 2.2. Electronic Components

Commercial oxygen sensors (CS) AP-0001 O2-A2 were purchased from AlphaSense (New York, NY, USA), Nanopower Operational Amplifiers TLV854x 500-Na were obtained from Texas Instruments (Dallas, TX, USA), and an Arduino Uno was used as the main processor for remote applications (Arduino, Italy). Printed circuit boards (PCBs) were designed and manufactured at our own facilities using FR-4 (KB-6150) as the substrate and two-layer technology.

### 2.3. Oxygen Sensor Design

**Figure 1:**
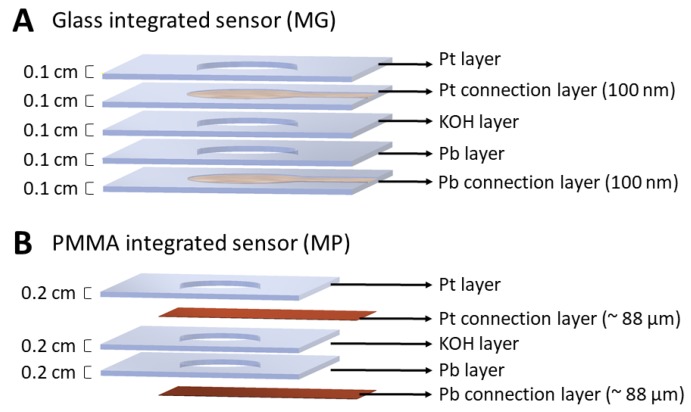
(**A**) Model of Glass-integrated sensor (MG); (**B**) Model of Polymethylmethacrylate (PMMA)-integrated sensor (MP).

Two different oxygen sensor designs were tested as potential models for further microsystem integration for compost measurements. Both designs were based on the use of platinum as the sensor cathode; KOH as the electrolyte for the semi-permeable membrane; and lead as the sensor anode. AlphaSense AP-0001 O2-A2 sensors were used as the control for all experiments. The model of glass-integrated sensor (MG) was designed based on the incorporation of discrete elements, glass as substrate, and was composed of five layers (Figure 1A). The bottom layer was made of a conductive adhesive tape to connect the anode. On top of this layer, a small pellet of lead (~30 mg) was placed inside a perforated glass slide (1 mm thickness). Another perforated glass slide was placed on top including a paper membrane soaked in KOH. Finally, the top layers included platinum connected to another conductive adhesive tape and a perforated glass slide used as the top cover. The manufacture of the perforated glass slides was carried out by wet chemical etching of the glass using 40% HF and standard photolithography.

The model of PMMA-integrated sensor (MP), also based on the incorporation of discrete elements, presented the same structure described above, but the substrate material was substitute by PMMA with an area of 75 mm × 25 mm and 2 mm thickness (Figure 1B). In these sensors, photolithography and wet chemical etching was substituted by laser-cutting of PMMA which eased the manufacturing process and reduced the cost of materials and equipment used during the process.

### 2.4. Oxygen Sensor Characterizations

Oxygen sensors were monitored by measuring the output voltage over 2 h. Sensors were placed inside a hermetic chamber under a nitrogen atmosphere. Hereafter, a constant volume of air was injected per cycle during multiple cycles to determine the electrical response of the sensors and their instrumental circuit to an increment of the oxygen concentration. A commercial oxygen sensor (CS) was used as the control in all experiments.

### 2.5. Sensor Lifetime

Selected oxygen sensor models, according to their performance, and a commercial oxygen sensor were monitored by measuring the output voltage over 12 h to determine their lifetime. Voltage corresponded to the current produced by the sensor connected to a 100 Ω resistance between its terminals. The sensors were exposed to dry air and were connected to the acquisition system during the whole process. All experiments were carried out using two independent sensors compared to the commercial one. The sampling rate for each sensor was 1 measurement per second.

### 2.6. Integration to Respirometric Microsystem

**Figure 2:**
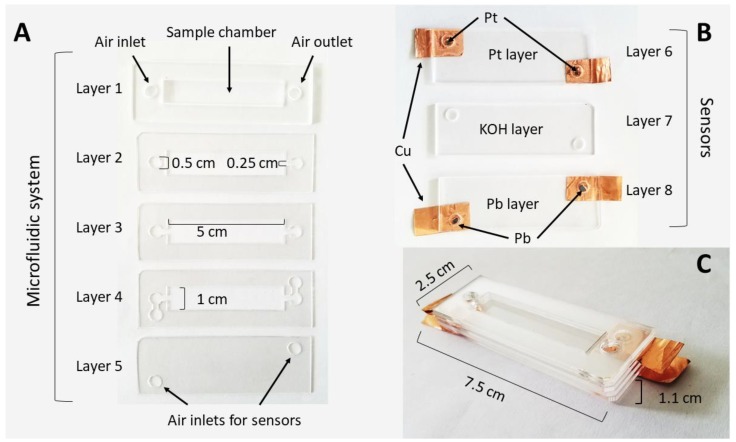
Respirometric microsystem description. (**A**) Microfluidic system; (**B**) oxygen sensors; (**C**) respirometric microsystem.

Selected sensors were integrated into a microfluidics system to create an integrated respirometric microsystem. These devices were used for further testing as a method for determining the respirometry response of static and dynamic processes. The respirometric microsystem was composed of two main parts: the microfluidic system and oxygen sensors. The microfluidic system consisted of a sample chamber and air inlets and outlets with five PMMA layers (layers 1 to 5), 75 mm × 25 mm and 1 mm of thickness. All manufactured layers were laser cut and pasted with methylene chloride. Each layer was characterized by different geometrical structures that allowed the flow of air in a specific direction through the chambers (Figure 2A). The MP sensors were selected as the oxygen sensors to be integrated, as described in the Results section. Two oxygen MP sensors (layers 6 to 8) were located under the microfluidic system and were connected through layer 5 (Figure 2B). The microfluidic chamber and sensors were assembled using methylene chloride. The final respirometric microsystems consisted of 75 mm × 25 mm × 11 mm structures (Figure 2C).

### 2.7. Instrumental Circuit Design

**Figure 3:**
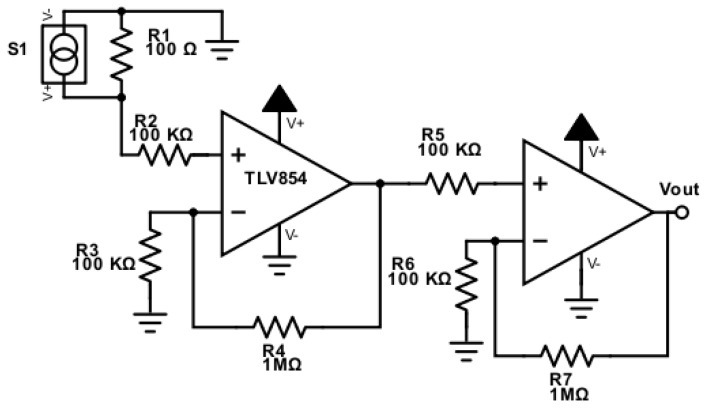
Schematic of the amplification circuit.

Oxygen sensors were coupled to an electronic circuitry to amplify the signal. Figure 3 shows the two-stage amplifier configuration with TLV854x 500-Na OpAmps. The oxygen sensor acted as an oxygen-powered current source that was converted to a voltage signal and amplified 100 times for an output signal ranging from 0 to 1.2 V.

### 2.8. Static Respirometry Measurements of Compost 

Compost was prepared with garden waste and organic matter with a proportion of 1:10, respectively, inside a rotary drum of 220 L. Compost analysis was monitored through temperature and respirometry measurements one time per week for two months. This monitoring frequency was selected according to different authors [13,26,42] related to the time the process takes to present significant variations. In addition, the commercial Ecozone System, used as a reference, took 24 h and a great amount of sample, that had to be discarded, to complete each measurement. In this sense, the Ecozone System limited the sampling rate in the experimentation. Temperature was measured directly in the compost using a thermocouple K, and it was calculated as the average of three measurements distributed at the mid height of the drum reactor. Respirometry was determined according to ISO 16072:2011 by analyzing the mixture of three independent samples taken from the mid height of the drum reactor. The mixture was analyzed by static respirometry measurement (SRI) using the respirometric microsystem and a commercial system named Ecozone. The EcoZone System ME-6668 (Pasco, USA) was composed of a hermetic chamber and a luminescence PASPORT Oxygen Gas Sensor. The system also included a SPARK LXi Datalogger for registering the measurements. The SRI was also performed in the respirometric microsystem by placing the sample in a hermetic chamber with 1/5 of the volume used for the EcoZone system. A CS was also incorporated in the hermetic chamber for the SRI test when using the respirometric microsystem as a control measurement. All measurements related to the respirometric microsystem were carried out by duplicates.

### 2.9. Dynamic Respirometric Measurement of the Compost

The same mixture of compost, used for the SRI after six weeks, was used for dynamic respirometric measurement (DRI) when the compost was more stable [18,24,43].The DRI was carried out in duplicate and consisted of the injection of a continuous airflow through the sample in the respirometric microsystem over 90 min [44]. The inflow of dry air was monitored with the oxygen sensor MP, already integrated into the microsystem, while the outflow was monitored with an outlet MP integrated sensor and an AlphaSense commercial oxygen sensor as a control. 

### 2.10. Cost Analysis

Cost analysis was performed for the two oxygen sensor designs (i.e., MG and MP) and for three respirometric methods: one commercial, one standard laboratory test, and one from the present study (i.e., EcoZone System, titration, and respirometric microsystem). 

The oxygen sensor cost analysis was calculated based on the cost of materials and manufacture processes involved, as described in Equation (1). Most of the costs of the reagents and compounds were obtained from MilliporeSigma (St. Louis, MO, USA). Other unitary prices were obtained from the local market. Nevertheless, the cost of manpower was not considered in the analysis, since it depends on the automation and monitoring of the process and the experience of the worker.

(1) $$C_{sensor}=\sum^{\;}\left(C_{Mt}\times U_{Mt}\right)+\sum^{\;}\left(C_{Mf}\times U_{Mf}\right)$$

where, *C_sensor_* corresponds to the total cost of the sensor; *C_Mt_* and *U_Mt_* are the cost of the raw material and the amount used; and *C_Mf_* and *U_Mf_* are the cost of the manufacture processes and the number of processes involved.

The cost analysis for the respirometric methods consisted of the sum of material cost (CM), equipment cost (*C_Eq_*), and operating and manpower cost (*C_Op_*) (Equation (2)) [45]. The commercial EcoZone System presented no material costs, as the equipment does not require the use of reagents. The material cost for the respirometric microsystem was based on the cost of the manufactured sensors and microsystem, calculated as described in Equation (1). The cost of the equipment was calculated taking into consideration the lifetimes of each unit—five years—and the average use of each unit during its lifetime. Operating and manpower cost was calculated according to the standard fees of technicians and operators.

(2) $$C_{Total}=C_{M}+C_{Eq}+C_{Op}$$

### 2.11. Analytic Hierarchy Process

An analytic hierarchy process (AHP) was performed in order to study the viability of the designed oxygen sensors compared to the AlphaSense commercial sensor. Another AHP was carried out to study the viability of the manufactured respirometric microsystem compared to two available measuring methods, the commercial EcoZone System and a standard laboratory test of titration. The criteria were selected based on previous studies that applied AHPs to analyze sensors, laboratory methods or technological approaches [46,47,48,49,50]. Matrix pairwise comparisons were constructed with the Scale of Saaty, based on information obtained from local experts and laboratories. The AHP of the oxygen sensors were evaluated based on five criteria: stability, scalability of the manufacturing process, sensibility, cost, and manufacturing time. Each criterion was evaluated with a score between 0 and 10, where 0 was an undesired scenario and 10 was an optimal scenario. Stability referred to the performance of the sensor over time. Scalability of the manufacturing process described the possibility of manufacturing the sensors in high yield. Sensitivity referred to the change in the output signal according to a change in the stimulus of the sensor. Cost referred to the cost of the sensors described in Section 2.10. Manufacturing time referred to the time that it takes to manufacture a type of sensor. 

In a similar way, respirometric methods of AHPs were evaluated based on five criteria: cost, portability, accuracy, analysis time, and integration of new technologies. These criteria were evaluated with a score between 0 and 10, where 0 was an undesired scenario and 10 was an optimal scenario. Cost was the result of the respirometric methods cost analysis described in Section 2.10. Portability referred to the requirements of facilities and the ease of mobilization. Accuracy referred to the difference between measured and true values. Analysis time was the time it takes to analyze each sample and obtain a stable measurement. Finally, integration of new technologies implies the possibility of the respirometric method equipment to communicate with other measurements or IoT equipment. In both AHPs, the matrix pairwise comparison process was evaluated using the consistency index defined by Saaty [51]. 

## 3. Results and Discussion

### 3.1. Oxygen Sensor Design

The MG oxygen sensor presented smaller dimensions and a lower cost of materials than the AlphaSense commercial sensor. However, the MG oxygen sensor was compose of many glass-based layers and, therefore, the photolithography process and HF wet chemical etching needed for its production was prolonged. This process needed sophisticated equipment and specialized manpower. It was difficult to maintain copper connections with lead because the HF etching removed necessary copper areas; also, it was difficult to line up the copper connection of the layers. The glass substrate was compatible with the biological samples that were analyzed, but it was not the best suitable substrate because of its high fragility and the possibility of it being broken during the manufacturing process.

The MP oxygen sensor presented smaller dimensions than the commercial sensor, and its cost of materials and manufacturing processes were low compared to the other sensors. The PMMA substrate was more resistant than the glass substrate during the manufacture of the sensor structure, presenting no fractures at any stage of the process. The manufacturing process did not require highly trained personnel, and laser cutting technology reduced its manufacturing time compared to the wet chemical etching processes used for the fabrication of the other type of sensors. Thus, this manufacturing process can be easy replicated at the industrial scale. 

### 3.2. Oxygen Sensor Characterization

**Figure 4:**
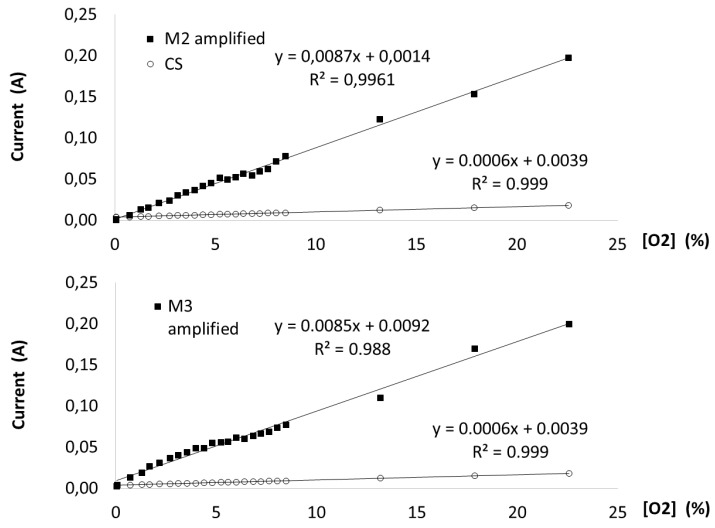
The MG and MP oxygen sensor characterizations.

The two oxygen sensors were characterized using different oxygen concentrations and compared to an AlphaSense commercial sensor. The electrical signals obtained from the sensors were amplified, as described previously. Both sensors’ responses towards oxygen concentrations revealed a comparable signal to the AlphaSense commercial sensor signal, with a lineal response and regression coefficients greater than 0.988, as shown in Figure 4. The MG and MP oxygen sensors presented an adequate stability in the measurement of oxygen concentration between 0% and 21%. However, for the further tests of characterization, lifetime analysis, and respirometry, the MP was selected as the model sensor due to the signal stability presented, cost of production related to the cost analysis, and the possibility of scaling up its production.

### 3.3. Sensor Lifetime

**Figure 5:**
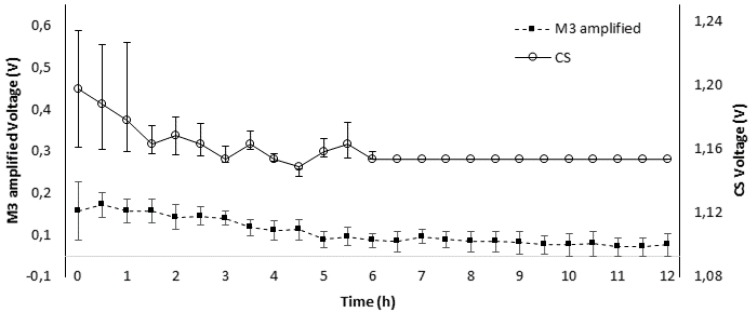
The MP oxygen sensors’ lifetime.

The lifetime of MP sensors was tested by exposing it to dry air for 12 h while being monitored with the acquisition system during the whole process. In addition, a commercial sensor was also included as a reference. Each sensor was monitored with a sampling rate of 1 measurement per second during the test. Figure 5 illustrates a simplification of the data, showing the average measurement each 30 min. The MP sensors presented a high variability in early life, also presented at the commercial sensor. The sensor signals decreased with a significant slope between 1 and 5 h. Subsequently, the rate of decline was stable between 6 and 12 h. The sensor signals lost stability among replicates, approximately after 8 h. Thus, the MP sensors’ lifetimes were calculated on average as 8 h, but it was highly dependent on the exact number of initial components placed during the manufacturing. Therefore, the most relevant parameters on the sensor lifetime are related to the amount of Pb, KOH, and Pt and the adequate conditions to keep the KOH in the sensor.

### 3.4. Static Respirometry Measurements (SRI) of Compost 

**Figure 6:**
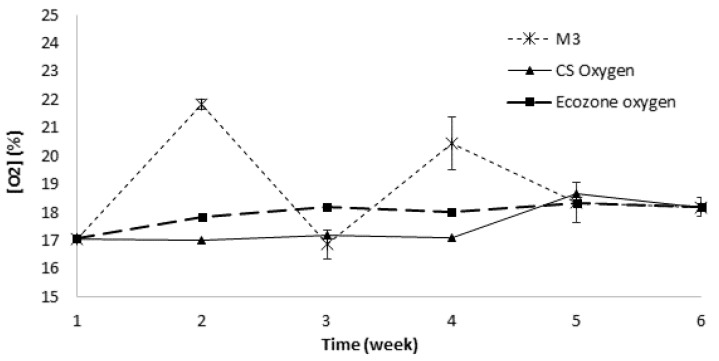
Static respirometry profiles.

Static respirometry profiles for EcoZone System, the AlphaSense commercial sensor, and the respirometric microsystem using MP are presented in Figure 6. The EcoZone System reported stable measures of oxygen consumption until the fourth week, and a slight increase of 1% during the fifth week. The AlphaSense commercial sensor also reported stable measures over the six weeks similar to that measured by the EcoZone System. In contrast, the respirometric microsystem presented a greater variability in the measurements. This may have been due to the higher sensibility of the oxygen consumption changes during the compost process. Other authors [18] have reported slight oxygen consumption changes, presuming a higher sensitivity of the respirometric microsystem. Oxygen consumption did not occur at the beginning of the process; however, oxygen began to be consumed until the level of oxygen decreased causing the death of aerobic microorganisms. Consequently, the consumption of oxygen decreased again, and the level of oxygen increased. This process can be easily identified as a cycling process, with a tendency to be stabilized by the end of the process [18,24,43,52], matching the oxygen consumption of the other two techniques. Therefore, the respirometric microsystem profile could evidence an oxygen consumption trend related to that previously reported by different authors.

### 3.5. Dynamic Respirometry Measurement (DRI) of Compost

**Figure 7:**
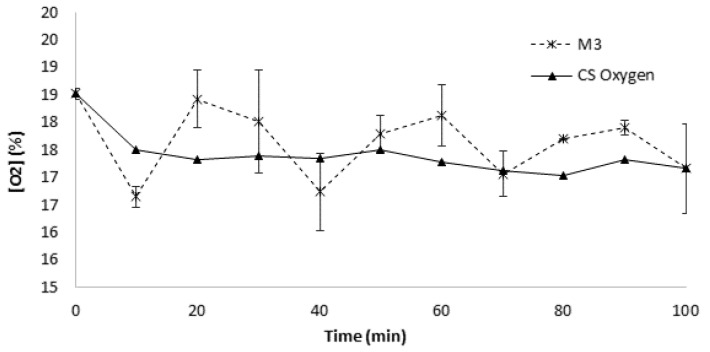
Dynamic respirometry profiles.

The dynamic respirometry profiles are represented in Figure 7 as a function of time with the injection of a continuous airflow. All DRI measurements ranged from 16.5 to 18.5% of O_2_, below 19%, which, according to the European Committee for Standardization (CEN), is a stability limit for composts [53,54]. The compost can be considered stable after 6 weeks during the process. The DRI measurements in this study, both for the commercial sensor and for the MP-based microsystem, reported similar behaviors, once again illustrating a higher sensitivity from the respirometry microsystem showing a cycling behavior. Other authors have reported similar decreasing trends in the DRI measurements of compost [53,54].

### 3.6. Cost Analysis

Table 1 summarizes the costs for the MG and MP oxygen sensor designs. The MP sensors could be considered low-cost sensors compared to the AlphaSense commercial sensor, with a cost of less than one-tenth of the cost of the commercial one. Moreover, the cost of the MG oxygen sensor was four times the cost of the AlphaSense commercial sensor. In this sense, MG was a high-cost oxygen sensor, minimizing their viability to be mass produced. Table 2 summarizes the costs for the three respirometric methods. The commercial EcoZone System was the most expensive one with a cost of 34 USD, followed by the titration standard test with a cost of 13 USD. Finally, the respirometric microsystem presented a cost of only 6 USD. 

### 3.7. Analytic Hierarchy Process (AHP) 

**Figure 8:**
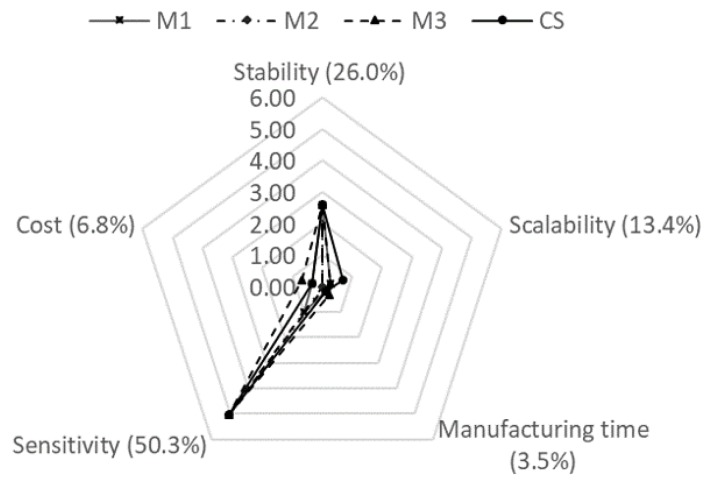
Radar chart for oxygen sensors scores with respect to the analytic hierarchy process criteria.

**Figure 9:**
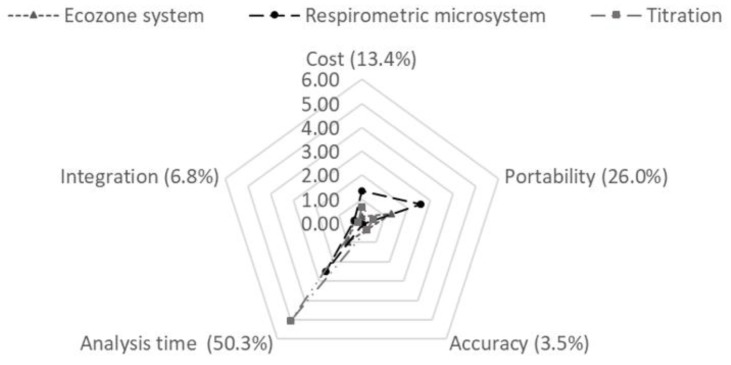
Radar chart for respirometric methods scores with respect to the analytic hierarchy process criteria.

Based on the matrix pairwise comparison process, different weights which represented the dominance of each criterion were obtained for the oxygen sensor designs and respirometric methods. The weights obtained in the AHP for the oxygen sensor designs were 26% for the stability, 13.4% for the scalability of the manufacturing process, 3.5% for the manufacturing time, 50.3% for the sensitivity, and 6.8% for the cost. The MP oxygen sensor was selected as the best design because it obtained the highest overall score from the studied design in this work of 8.93; while the MG presented a global score of 7.70. The AlphaSense commercial sensor, used as a control, presented a score of 8.82 (Figure 8). The weights obtained in the AHP for the respirometric methods were 13.4% for the cost, 26% for the portability, 3.5% for the accuracy, 50.3% for the analysis time, and 6.8% for the integration of new technologies. The order of selection for the respirometric methods based on global scores was respirometric microsystem (6.87), titration (6.70), and EcoZone system (3.26) (Figure 9).

## 4. Conclusions

Based on the characterization, oxygen sensor manufacturing process, cost analysis, and analytic hierarchy process, the best proposed sensor was MP. This oxygen sensor design was based on the incorporation of discrete elements of PMMA substrate material and included laser cutting in the manufacturing process. The integration between MP sensor and the respirometric chamber did not require specialized manpower, the substrate was durable, and the scalability manufacturing process was feasible. The respirometric microsystem followed the oxygen consumption trends of a composting process under static and dynamic respirometric tests, evidencing slight cycling variations that tend to stabilize according to time on both type of tests. Based on the cost analysis and analytic hierarchy process of the respirometric methods compared in this study, it can be concluded that the respirometric microsystem is adequate for its use as an in situ method due to the fact of its cost and the facility of portability; however, it lacks both in accuracy and time, compared to the titration lab tests measurements. Nevertheless, the respirometric microsystem presented in this study allows the monitoring of the respirometry in a composting process in situ, shedding light on the integration of the monitoring of composting.

## Figures and Tables

**Table 1:** Oxygen sensor cost analysis under different designs.

Oxygen Sensor Design	Cost (USD)
MG	384
MP	2
CS	99

**Table 2:** Respirometric test cost analysis under different respirometric methods.

Respirometric Methods	Cost (USD)
Respirometric microsystem	6
EcoZone system	34
Titration	13
